# Development of DArT Marker Platforms and Genetic Diversity Assessment of the U.S. Collection of the New Oilseed Crop Lesquerella and Related Species

**DOI:** 10.1371/journal.pone.0064062

**Published:** 2013-05-28

**Authors:** Von Mark V. Cruz, Andrzej Kilian, David A. Dierig

**Affiliations:** 1 USDA-ARS National Center for Genetic Resources Preservation, Fort Collins, Colorado, United States of America; 2 Diversity Arrays Technology Pty. Ltd., Yarralumla, ACT, Australia; 3 Department of Bioagricultural Sciences and Pest Mgt., Colorado State University, Fort Collins, Colorado, United States of America; University of Illinois, United States of America

## Abstract

The advantages of using molecular markers in modern genebanks are well documented. They are commonly used to understand the distribution of genetic diversity in populations and among species which is crucial for efficient management and effective utilization of germplasm collections. We describe the development of two types of DArT molecular marker platforms for the new oilseed crop lesquerella (*Physaria* spp.), a member of the Brassicaceae family, to characterize a collection in the National Plant Germplasm System (NPGS) with relatively little known in regards to the genetic diversity and traits. The two types of platforms were developed using a subset of the germplasm conserved *ex situ* consisting of 87 *Physaria* and 2 *Paysonia* accessions. The microarray DArT revealed a total of 2,833 polymorphic markers with an average genotype call rate of 98.4% and a scoring reproducibility of 99.7%. On the other hand, the DArTseq platform developed for SNP and DArT markers from short sequence reads showed a total of 27,748 high quality markers. Cluster analysis and principal coordinate analysis indicated that the different accessions were successfully classified by both systems based on species, by geographical source, and breeding status. In the germplasm set analyzed, which represented more than 80% of the *P. fendleri* collection, we observed that a substantial amount of variation exists in the species collection. These markers will be valuable in germplasm management studies and lesquerella breeding, and augment the microsatellite markers previously developed on the taxa.

## Introduction

Lesquerella (*Physaria* sp.), a member of the Brassicaceae, is a North American genus which has more than 90 member species thriving mostly in dry and arid habitats usually mixed with sparse vegetation [1], [2]. Commonly known as bladderpod, it has been identified by the U.S. Department of Agriculture and the U.S. Department of Energy as very promising species for the production of lubricants, engine oils, waxes, coatings and a source of natural estolides with valuable application in the automobile and biofuel industries [3], [4], [5]. There has been an increasing trend in research activities in the crop since its oil was first characterized for novel properties [6]. The fatty acid profile of lesquerella oil has been found to vary. Species found east of the Mississippi have mostly densipolic acid, those in the western U.S. mostly lesquerolic acid, and a species from Oklahoma predominantly with auricolic acid [7]. Lesquerella could be grown successfully as a winter annual in the southwest U.S. producing an average seed yield of 1.7 tons/ha [8]. At present, there are more than eight advanced breeding lines of *P. fendleri* ready for commercialization and a corresponding substantial germplasm collection has been assembled in the U.S. National Plant Germplasm System [9].

Molecular characterization of germplasm collections supplements phenotypic assessment of diversity and is important in the effective management of genetic resources. Molecular markers have been very useful in efforts to accurately identify gaps and redundancy within and among individual germplasm collections and have helped resolve important genebank management issues [10], [11], [12]. Examples of markers used in specific Brassicaceae collections include microsatellites for examining diversity in *B. napus* [13], [14], [15], lesquerella [16] and wild relatives such as *Capsella*, *Crambe* and *Sinapis* [17]; AFLPs in *B. oleracea* [18] and *Lepidium* [19]; and RAPD markers in *Raphanus* [20].

Compared to the previously mentioned molecular marker systems, Diversity Array Technology (DArT) markers are relatively new and were developed only in the early 2000. DArT markers overcame the difficulties in correlating bands on gels with allelic variants by utilizing hybridization-based methods and solid state surfaces [21]. They have been increasingly utilized as evidenced by the number of publications reporting successful implementation [22]. Among its advantages over other marker systems include high throughput capability allowing rapid germplasm characterization in a single experiment, independence of sequence data, and ability to detect single base changes and indels [23]. To date, DArT markers have been successfully applied in genetic diversity analysis, linkage mapping and in finding out population structure of collections in various crop species [24]. Their application in minor crops are likewise increasing due to their potential to accelerate gene discovery and initiate molecular breeding because of their whole genome coverage without relying on prior sequence data information [25], [26].

In lesquerella, allozymes have previously been used [27] and microsatellite markers have been developed [16]. But the small number of markers that are available presents a limitation in linkage mapping and in the study of genetic resources collections. At present, only fifteen microsatellites have demonstrated utility across different *Physaria* species. We present in this paper the development of two platforms of high density DArT markers for lesquerella and the results obtained after testing them to analyze the genetic diversity of *Physaria* and *Paysonia* national germplasm collection. This new molecular marker system for the new crop species will serve as an additional resource to augment the existing systems to assist crop improvement efforts, germplasm management activities and genetic studies.

## Materials and Methods

### Tissue Sampling

The lesquerella germplasm set was obtained from collections of the USDA-ARS National Arid Land Plant Genetic Resources Unit, Parlier, CA and the USDA-ARS Arid Land Agricultural Research Center, Maricopa, AZ. The samples used in this study were selected based on each accession’s geographic location, sampling within distinct counties in each State when possible. Seven advanced *P. fendleri* breeding lines, WCL-LH1, WCL-LO1, WCL-LO2, WCL-LO4, WCL-LY1, WCL-SL1 and WCL-YS1 were included among the samples. DNA was extracted from fresh leaf tissue obtained from five week old seedlings using Qiagen DNeasy 96 Plant Kits (Qiagen Inc., Valencia, CA). Two DArT platforms were developed for lesquerella as described below. The platform development utilized 86 *Physaria* accessions (see Table 1), representing 11 species and one *Paysonia* accession since the latter is a sister genus of *Physaria* and only recently was there a recognized taxonomic delineation between them [2].

**Table 1:** Passport information of accessions used in *Physaria* DArT and DArTseq platform development.

Collection No.and ID	Accession No.	Species	Country	State/Prov.	County	Latitude	Longitude	Elevation (m)
1809	W6 20822	*P. fendleri*	United States	AZ	Cochise	31.75600	−110.05500	134.15
1817	W6 20823	*P. fendleri*	United States	AZ	Cochise	31.74100	−109.96800	1493.90
1818	W6 20824	*P. fendleri*	United States	AZ	Cochise	31.50000	−110.00000	1432.56
1819	W6 20825	*P. fendleri*	United States	AZ	Santa Cruz	31.72400	−110.52600	1493.90
1826	W6 20856	*P. fendleri*	United States	AZ	Santa Cruz	31.72300	−110.52400	1463.41
1833	W6 20833	*P. gordonii*	United States	NM	Hidalgo	31.93333	−108.95000	1024.13
1834	PI 641918	*P. fendleri*	United States	NM	Hidalgo	31.93333	−108.93333	1292.35
1835	–	*P. fendleri*	United States	NM	Luna	32.21667	−107.41667	1277.11
1840	W6 20858	*P. fendleri*	United States	NM	Eddy	32.61667	−104.40000	1042.42
1841	PI 596417	*P. fendleri*	United States	NM	Eddy	32.31667	−104.25000	1130.81
1842	W6 20860	*P. fendleri*	United States	NM	Eddy	32.53333	−103.98333	1060.70
1843	PI 596418	*P. fendleri*	United States	NM	Eddy	32.75000	−104.10000	1082.04
1844	PI 596419	*P. fendleri*	United States	NM	Lincoln	33.68333	−105.83333	1636.78
1845	PI 596420	*P. fendleri*	United States	NM	Lincoln	33.68333	−105.91667	1572.77
1848	PI 596421	*P. fendleri*	United States	AZ	Graham	33.25000	−110.28333	795.53
1851	W6 20826	*P. fendleri*	United States	AZ	Cochise	31.91667	−109.16667	1493.52
1852	W6 20861	*P. fendleri*	United States	AZ	Cochise	31.65000	−110.01667	1447.80
1874	PI 596423	*P. fendleri*	United States	AZ	Apache	34.56667	−109.50000	1755.65
1889	PI 596425	*P. fendleri*	United States	NM	Socorro	33.88333	−106.40000	1661.16
1904	W6 20865	*P. fendleri*	United States	AZ	Apache	34.35000	−109.36667	1859.28
1906	PI 596426	*P. fendleri*	United States	NM	Socorro	34.41667	−106.65000	1645.92
1909	W6 20868	*P. fendleri*	United States	NM	Guadalupe	33.56667	−105.05000	1697.74
1910	PI 596428	*P. fendleri*	United States	NM	De Baca	34.46667	−104.30000	1149.10
1912	PI 596429	*P. fendleri*	United States	NM	Guadalupe	34.73333	−104.46667	1359.41
1919	PI 596430	*P. fendleri*	United States	NM	San Juan	36.60000	−108.91667	1758.70
1920	PI 596431	*P. fendleri*	United States	NM	San Juan	36.58333	−108.96667	1932.43
1924	W6 20851	*P. rectipes*	United States	AZ	Luna	35.86667	−109.60000	1912.00
1932	PI 596433	*P. fendleri*	United States	AZ	Graham	33.28333	−110.38333	877.82
2212	W6 20863	*P. argyraea*	United States	TX	Uvalde	29.15000	−99.70000	228.60
2226	W6 20828	*P. fendleri*	United States	TX	Webb	27.36667	−98.98333	243.84
2232	PI 643174	*P. lindheimeri*	United States	TX	Nueces	27.06667	−97.06667	107.00
2243	W6 20835	*Paysonia grandiflora*	United States	TX	Wilson	29.16667	−97.93333	91.44
2250	W6 20854	*P. recurvata*	United States	TX	Medina	29.53333	−99.21667	434.34
2255	PI 596435	*P. fendleri*	United States	TX	El Paso	31.83333	−106.01667	1341.12
2256	PI 596436	*P. fendleri*	United States	TX	Hudspeth	31.70000	−105.41667	1240.54
2260	PI 596439	*P. fendleri*	United States	TX	Hudspeth	31.81667	−105.13333	1219.20
2262	PI 596441	*P. fendleri*	United States	TX	Culberson	31.36667	−104.81667	944.88
2269	PI 596447	*P. fendleri*	United States	TX	Pecos	30.71667	−103.18333	975.36
2273	PI 596450	*P. fendleri*	United States	TX	Brewster	30.20000	−103.56667	1554.48
2277	PI 596452	*P. fendleri*	United States	TX	Brewster	29.78333	−103.18333	853.44
2278	PI 596453	*P. fendleri*	United States	TX	Brewster	30.20000	−103.20000	1310.64
2281	PI 596454	*P. fendleri*	United States	TX	Crockett	30.93333	−101.86667	640.08
2282	PI 596455	*P. fendleri*	United States	TX	Pecos	30.96667	−101.96667	563.88
2286	PI 596456	*P. fendleri*	United States	TX	Andrews	32.31667	−102.50000	807.72
2290	PI 596457	*P. fendleri*	United States	TX	Winkler	31.78333	−103.26667	853.44
2291	PI 596458	*P. fendleri*	United States	TX	Reeves	31.11667	−103.71667	944.88
2292	PI 596459	*P. fendleri*	United States	TX	Reeves	31.01667	−103.73333	1005.84
2294	PI 596461	*P. fendleri*	United States	TX	Jeff Davis	30.53333	−104.10000	1722.12
2295	PI 596462	*P. fendleri*	United States	TX	Jeff Davis	30.55000	−103.91667	1676.40
2296	PI 596463	*P. fendleri*	United States	TX	Jeff Davis	30.43333	−103.96667	1554.48
2299	PI 596464	*P. fendleri*	United States	TX	Presidio	30.15000	−104.08333	1402.08
2300	PI 596465	*P. fendleri*	United States	TX	Brewster	30.31667	−103.78333	1493.52
2301	PI 596466	*P. fendleri*	United States	TX	Presidio	30.26667	−103.90000	1432.56
2302	PI 596467	*P. fendleri*	United States	TX	Jeff Davis	30.53333	−104.31667	1493.52
2303	PI 596468	*P. fendleri*	United States	TX	Jeff Davis	30.71667	−104.66667	1280.16
2304	W6 20872	*P. fendleri*	United States	TX	Culberson	30.83333	−104.78333	1188.72
2305	W6 20830	*P. fendleri*	United States	TX	Hudspeth	31.03333	−104.90000	1310.64
2928	W6 20834	*P. gracilis*	United States	OK	Choctaw	34.01667	−95.35000	137.00
3009	W6 20319	*Paysonia auriculata*	United States	OK	Caddo	35.54173	−98.57041	475.00
4005	PI 643177	*P. fendleri*	Mexico	COA	–	28.73861	−100.91250	330.00
4008	–	*P. fendleri*	Mexico	COA	–	28.69328	−100.57672	270.97
4015	–	*P. fendleri*	Mexico	COA	–	25.11048	−101.11323	2097.00
4016	PI 643179	*P. fendleri*	Mexico	ZAC	–	24.37749	−101.38860	1840.00
4024	PI 641924	*P. fendleri*	Mexico	DUR	–	24.59139	−103.92303	1935.00
4042	–	*P. fendleri*	Mexico	COA	–	28.49582	−100.70158	312.72
4043	–	*P. fendleri*	Mexico	COA	–	28.53963	−100.48233	271.27
4044	–	*P. fendleri*	Mexico	ZAC	–	24.24713	−101.43585	1897.68
4045	–	*P. fendleri*	Mexico	ZAC	–	24.23212	−101.44367	1908.05
4046	–	*P. fendleri*	Mexico	ZAC	–	24.06105	−101.44598	1909.27
4047	–	*P. fendleri*	Mexico	NLE	–	25.10865	−100.65158	1919.94
4048	–	*P. fendleri*	Mexico	ZAC	–	24.21158	−101.45683	1935.18
4049	–	*P. fendleri*	Mexico	ZAC	–	24.39118	−101.45952	1933.35
4050	–	*P. fendleri*	Mexico	ZAC	–	24.19925	−101.46575	1930.91
4058	–	*P. fendleri*	United States	NM	McKinley	36.44348	−108.13162	2232.66
4085	–	*P. fendleri*	United States	NM	San Miguel	35.30000	−104.25385	1368.00
4087	–	*P. kathyrn*	United States	ME	–	–	–	–
4092	PARL 812	*P. pallida* *	United States	TX	–	–	–	–
4094	PARL 814	*P. pallida* *	United States	TX	–	–	–	–
4057b	–	*P. fendleri*	United States	NM	McKinley	–	–	–
DDMC2010-1	PARL 859	*P. fendleri*	United States	NM	Socorro	34.13115	−106.90907	1424.00
DDMC2010-19	PARL 874	*P. fendleri*	United States	TX	Jeff Davis	30.79047	−104.16759	1668.00
DDMC2010-6	PARL 863	*P. gordonii*	United States	TX	Hudspeth	31.74230	−105.10552	1141.00
WCL-LO2	–	*P. fendleri*	United States	–	–	–	–	–
WCL-LO4		*P. fendleri*	United States	–	–	–	–	–
WCL-SL1	PI 613132	*P. fendleri*	United States	–	–	–	–	–
WCL-YS1	PI 610492	*P. fendleri*	United States	–	–	–	–	–
–	PARL 817	*P. thamnophila*	United States	TX	–	–	–	–

* exact geographic coordinates of *P. pallida* are not supplied since they are in the federal and state list of endangered species.

### Microarray DArT Platform Development

The microarray DArT was developed by first testing combinations of the rare-cutting restriction enzyme *PstI* with several restriction endonucleases that cut frequently on DNA samples from 8 representative accessions to determine the restriction enzyme combination that provided the best complexity reduction. Final genomic representations were prepared using *PstI/BstNI* combinations. Approximately 50 ng of genomic DNA was digested with *PstI*/*BstNI* combinations and the resulting fragments ligated to a *PstI* overhang compatible oligonucleotide adapter. A primer annealing to this adapter was used in PCR reaction to amplify complexity-reduced representation of a sample. Amplification products were either used for cloning a in marker development process or labeled with fluorescent dyes and hybridized to DArT array in the genotyping process. Library construction was subsequently performed using 80 *P. fendleri* accessions and 16 accessions of wild related *Brassica* species. The amplified *PstI* restriction fragments from all accessions were cloned into pCR2.1-TOPO vector (Invitrogen, Australia) as described by Jaccoud et al. [21] and four libraries were generated. The white colonies containing genomic fragments inserted into pCR2.1-TOPO vector were picked into individual wells of 384-well microtiter plates filled with ampicillin/kanamycin-supplemented freezing medium. A total of 3,456 clones from *Physaria* were obtained –1,920 from three libraries of *P. fendleri* and 1,536 from the related species. Inserts from these clones were amplified using M13F and M13R primers in 384 plate format, a subset of PCR products were assessed for quality (10% of 25 µl PCR reaction) through gel electrophoresis, and all remaining PCR products dried, washed and dissolved in a spotting buffer. A total of 6,144 clones were printed with spot duplication on SuperChip poly-L-lysine slides (Thermo Scientific, Australia) using a MicroGrid arrayer (Genomics Solutions, UK). The microarrays included 1,920 *Brassica* clones and 768 *Arabidopsis* clones in addition to the *Physaria* clones.

Each sample was assayed using methods described above for library construction. Genomic representations were assessed for quality through gel electrophoresis in 1.2% agarose and labeled with fluorescent dyes (Cy3 and Cy5). Labelled targets were then hybridized to printed DArT arrays for 16 hours at 62°C in a water bath. Slides were washed as described by Kilian et al. [28], dried initially by centrifugation at 500 × g for 7 min and later by a desiccator under vacuum for 30 min. The slides were scanned using Tecan LS300 scanner (Tecan Group Ltd, Männedorf, Switzerland) generating three images per array: one image scanned at 488 nm for reference signal measures the amount of DNA within the spot based on hybridization signal of FAM-labeled fragment of a TOPO vector multiple cloning site fragment and two images for “target” signal measurement: one scanned at 543 nM (for Cy3 labeled targets) and one at 633 nM (for Cy5 labeled targets). All DArT techniques applied for this work were recently described in much more detail by Kilian et al. [28].

### DArTseq Platform Development

For the sequencing-based DArT genotyping, four complexity reduction methods optimized for several other plant species at DArT P/L were used. *PstI*-RE site specific adapter was tagged with 96 different barcodes enabling encoding a plate of DNA samples to run within a single lane on an Illumina Genome Analyzer IIx (Illumina Inc., San Diego, CA). *PstI* adapter included also a sequencing primer site, so that the tags generated were always reading into the genomic fragments from the *PstI* sites. After the sequencing run, the FASTQ files were quality filtered using the threshold of 90% confidence for at least 50% of the bases and in addition filtered more stringently for barcode sequences. Two lanes of GAIIx were run with all samples providing fully replicated sequencing data. The filtered data were split into their respective target (individual) data using barcode splitting script. Each sample had on average 500,000 counts per replicate. After producing various QC statistics and trimming of the barcode the sequences were aligned against the reference created from the tags identified in the sequence reads generated from all the samples. In addition the short sequence tags were aligned against *Arabidopsis thaliana*’s genome available in Genbank. *Arabidopsis* is a close relative of *Physaria* in the Brassicaceae [29]. The output files from alignment generated using Bowtie software [30] were processed using an analytical pipeline developed by DArT P/L to produce “DArT score” tables and “SNP” tables.

### Genotyping

Both DArT on array platform and DArTseq use a set of quality parameters to select markers which are of use for a specific application. One of these parameters is reproducibility of markers in technical replicates for a subset of samples. In diversity analysis, the reproducibility parameter threshold is set usually at 97% which translates to average reproducibility of the dataset around 99.7%. A total of 87 common accessions were genotyped using both the microarray DArT and DArTseq platforms. A total of 11 *Physaria* species, 1 interspecific hybrid, and 2 *Paysonia* species were analyzed by microarray DArT. Additional accessions were genotyped using the DArTseq platform comprised of 17 *Physaria* and 7 *Paysonia* species. Overall, there was a total of 177 accessions represented by single plant samples genotyped using the two platforms, majority of which are *P. fendleri* (Tables 1 and 2). The marker sequences and genotype data will be stored in the U.S. Germplasm Resources Information Network (GRIN) database (http://www.ars-grin.gov) along with the accessions’ phenotypic observations and germplasm passport data curated by the U.S. National Plant Germplasm System.

**Table 2:** Passport information of additional *Physaria* and *Paysonia* accessions genotyped using DArTseq.

Collection No.and ID	Accession No.	Species	Country	State/Prov.	County	Latitude	Longitude	Elevation (m)
715	PI 293027	*P. fendleri*	United States	TX	Brewster	29.32768	−103.20527	1139.00
720	PI 293028	*P. fendleri*	United States	TX	Val Verde	29.84683	−101.68152	519.00
Barclay 998	PI 279650	*P. fendleri*	United States	NM	–	–	–	–
1836	W6 20857	*P. fendleri*	United States	NM	Luna	32.23333	−107.38333	1264.92
1838	PI 596415	*P. fendleri*	United States	NM	Chaves	32.86667	−105.03333	1621.54
1839	PI 596416	*P. fendleri*	United States	NM	Eddy	32.83333	−104.80000	1301.50
1854	W6 23374	*P. gordonii*	United States	AZ	Cochise	31.85000	−109.96667	1732.79
1855	PI 596422	*P. fendleri*	United States	AZ	Cochise	31.96667	−109.46667	1548.38
1871	W6 20862	*P. fendleri*	United States	AZ	Navajo	34.88333	−110.11667	1546.86
1879	W6 20838	*P. intermedia*	United States	AZ	Navajo	34.35000	−110.16667	1828.80
1880	PI 596424	*P. fendleri*	United States	AZ	Navajo	34.36667	−110.15000	1825.75
1902		*P. kaibabensis*	United States	AZ	Coconino	36.73333	−112.11667	2237.23
1907	PI 596427	*P. fendleri*	United States	NM	Torrance	34.55000	−106.13333	1850.14
1926	PI 596432	*P. fendleri*	United States	AZ	Navajo	34.75000	−110.16667	1615.44
1934	W6 20869	*P. fendleri*	United States	AZ	Graham	33.23333	−110.25000	908.30
2097	PI 331165	*P. fendleri*	United States	AZ	Graham	33.38333	−110.38333	917.00
2202	W6 20819	*P. densiflora*	United States	TX	Gillespie	30.20000	−98.93333	434.34
2203		*P. recurvata*	United States	TX	Kerr	30.00000	−99.36667	518.16
2217	PI 643172	*Paysonia lasiocarpa*	United States	TX	Frio	28.63333	−99.38333	198.12
2228	W6 20840	*Paysonia lasiocarpa*	United States	TX	Duval	27.58333	−98.56667	137.16
2246	W6 20836	*Paysonia grandiflora*	United States	TX	Atascosa	28.85000	−98.35000	121.92
2247	W6 20837	*Paysonia grandiflora*	United States	TX	Frio	29.06667	−98.85000	121.92
2257	PI 596437	*P. fendleri*	United States	TX	Hudspeth	31.73333	−105.21667	1097.28
2258	W6 20829	*P. fendleri*	United States	TX	Hudspeth	31.83333	−105.20000	1219.20
2259	PI 596438	*P. fendleri*	United States	TX	Hudspeth	31.81667	−105.13333	1188.72
2264	W6 20870	*P. fendleri*	United States	TX	Reeves	30.91667	−103.78333	914.40
2265	PI 596443	*P. fendleri*	United States	TX	Jeff Davis	30.86667	−103.78333	1127.76
2267	PI 596445	*P. fendleri*	United States	TX	Jeff Davis	30.68333	−103.78333	1234.44
2268	PI 596446	*P. fendleri*	United States	TX	Jeff Davis	30.48333	−103.75000	1554.48
2270	PI 596448	*P. fendleri*	United States	TX	Brewster	30.38333	−103.58333	1402.08
2274		*P. fendleri*	United States	TX	Brewster	30.03333	−103.56667	1280.16
2276	W6 20871	*P. fendleri*	United States	TX	Brewster	29.66667	−103.11667	822.96
2279	W6 20844	*P. mcvaughiana*	United States	TX	Pecos	30.46667	−102.91667	1432.56
2293	PI 596460	*P. fendleri*	United States	TX	Jeff Davis	31.00000	−104.18333	1310.64
2297	W6 20859	*P. fendleri*	United States	TX	Presidio	30.28333	−104.01667	1463.04
2401		*P. douglasii*	United States	WA	Grant	46.63333	−119.73333	89.61
2819	W6 20873	*P. fendleri*	–	–	–	–	–	–
2894		*Paysonia densipila*	United States	TN	Williamson	35.82861	−86.69722	208.00
2997	W6 20859	*P. fendleri*	United States	AZ	Apache	34.48333	−109.51667	1899.00
3029		*P. intermedia*	United States	UT	Garfield	38.02044	−112.36819	1944.00
3011	W6 20320	*Paysonia auriculata*	United States	OK	Blaine	35.79325	−98.42029	454.00
3000		*Paysonia lyrata*	United States	AL	Colbert	–	–	–
3030	PI 337050	*P. fendleri*	United States	NM	Sierra	33.36355	−107.28041	1500.00
3042	W6 20843	*P. ludoviciana*	United States	UT	Garfield	37.76753	−112.06660	1791.00
3050		*P. acutifolia*	United States	UT	San Juan	37.50160	−109.64779	1410.00
3058	PI 345712	*Paysonia auriculata*	United States	OK	Garfield	36.43716	−97.58647	360.00
3083	PI 355037	*P. fendleri*	United States	TX	Kinney	29.15197	−100.38664	288.00
3085		*P. acutifolia*	United States	CO	Mesa	38.83627	−108.56218	2073.00
3091	PI 355041	*Paysonia perforata*	United States	TN	Wilson	36.23255	−86.38455	182.00
3092	PI 355042	*Paysonia stonensis*	United States	TN	Rutherford	35.98204	−86.41221	158.00
319b		*P. argyraea*	–	–	–	–	–	–
3219	W6 20847	*P. pallida*	United States	TX	–	–	–	–
3370	PI 275769	*Paysonia lyrata*	United States	AL	Franklin	–	–	–
3344	PI 643175	*P. mexicana*	Mexico	COA	–	27.03333	−102.30000	1500.00
3347	PI 275771	*Paysonia stonensis*	United States	TN	Rutherford	35.99628	−86.44516	172.00
4001		*P. fendleri*	Mexico	COA	–	27.59103	−101.18092	394.41
4002	PI 643176	*P. fendleri*	Mexico	COA	–	28.13173	−101.12267	396.54
4006		*P. fendleri*	Mexico	COA	–	28.87570	−100.61240	304.80
4007	PI 641923	*P. fendleri*	Mexico	COA	–	28.61252	−100.50212	277.67
4014	PI 643178	*P. argyraea*	Mexico	COA	–	25.16667	−101.00000	2500.00
4055		*P. intermedia*	United States	AZ	Navajo	34.43717	−110.53613	2233.23
4061		*P. fendleri*	United States	–	–	–	–	–
4063		*P. inflata*	Mexico	NLE	–	25.64967	−100.70617	1303.96
4064		*P. fendleri*	United States	–	–	–	–	–
4088		*P. valida*	United States	NM	Otero	32.94567	−105.35584	1821.00
4091	PARL 811	*P. pallida*	United States	TX	–	–	–	–
4093	PARL 813	*P. pallida*	United States	TX	–	–	–	–
19224	PI 293005	*P. fendleri*	United States	TX	Val Verde	30.53333	−102.15000	500.00
19230	PI 293008	*P. fendleri*	United States	TX	Loving	32.23333	−103.90000	861.28
19231	PI 283700	*P. fendleri*	United States	TX	Loving	31.73413	−103.55457	855.00
19239	PI 293013	*P. fendleri*	United States	TX	Reeves	32.35000	−104.35000	1000.30
19242	PI 293015	*P. fendleri*	United States	TX	Reeves	32.06667	−103.86667	861.28
19992	PI 293016	*P. fendleri*	United States	TX	Hudspeth	32.26667	−105.61667	1300.30
20634	PI 299412	*P. fendleri*	United States	AZ	Conchise	31.83791	−110.34341	1386.00
WCL-LO1	PI 596363	*P. fendleri*	–	–	–	–	–	–
WCL-LY1	PI 596362	*P. fendleri*	–	–	–	–	–	–
WCL-LH1	PI 596364	*P. fendleri*	–	–	–	–	–	–
DDMC2010-2	–	*P. fendleri*	United States	NM	Lincoln	33.68458	−105.91996	1598.00
DDMC20103	PARL 860	*P. fendleri*	United States	NM	Lincoln	33.73022	−105.96510	1666.00
DDMC2010-4	PARL 861	*P. fendleri*	United States	NM	Eddy	32.91352	−104.43478	1041.00
DDMC2010-5	PARL 862	*P. fendleri*	United States	TX	Loving	31.73557	−103.55061	855.00
DDMC2010-7	PARL 864	*P. gordonii*	United States	NM	Dona Ana	32.25027	−107.17893	1324.00
DDMC2010-8	PARL 865	*P. fendleri*	United States	NM	Dona Ana	32.24291	−107.18271	1319.00
DDMC2010-9	PARL 866	*P. fendleri*	United States	AZ	Conchise	31.51705	−110.01781	1447.80
DDMC2010-10	PARL 867	*P. fendleri*	United States	AZ	Conchise	31.81811	−110.01729	1458.47
DDMC2010-11	PARL 868	*P. fendleri*	United States	AZ	Conchise	31.83494	−110.01163	1505.10
DDMC2010-12	PARL 869	*P. fendleri*	United States	TX	Culberson	30.93663	−104.81040	1207.92
DDMC2010-13	PARL 870	*P. fendleri*	United States	TX	Presidio	30.30604	−104.03443	1444.45
DDMC2010-14	–	*P. fendleri*	United States	TX	Brewster	30.50022	−103.44561	1200.91
DDMC2010-15	PARL 871	*P. fendleri*	United States	TX	Pecos	30.90170	−102.91230	929.34

* exact geographic coordinates of *P. pallida* are not supplied since they are in the federal and state list of endangered species.

### Data Analysis

All the images from DArT platforms were analyzed using DArTsoft v.7.4.7 (DArT P/L, Canberra, Australia). The markers were scored as binary data (1/0), indicating presence or absence of a marker in genomic representation of each sample as described by Wenzl et al. [31]. For quality control, 30% of genotypes were genotyped in full technical replication. Clones with P>77%, a call rate >97% and >98% allele-calling consistency across the replicates were selected as markers. P value represents the allelic-states variance of the relative target hybridization intensity as a percentage of the total variance. The informativeness of the DArT markers was determined by calculating the polymorphism information content (PIC) within the panel of diverse accessions according to Anderson et al. [32]. The *P. fendleri* data were used in GenAlEx v.6.41 [33] to determine 2D spatial autocorrelation of the DArT microarray and Arlequin v.3.5.1.3 [34] to assess the amount of variation among the assigned regional groupings by AMOVA. To summarize the relationships among all examined accessions, cluster analysis was performed on Dice similarity values with the SAHN procedure using the unweighted pair-group method done using NTSYS-pc v. 2.21 m [35]. The Dice coefficient was preferred over simple matching coefficient because DArT is a dominant marker system and there were several non-*Physaria* species in the sample set [36], [37]. In addition, a Bayesian model-based clustering was performed on microarray DArT markers using STRUCTURE v.2.3.4 [38] testing 3 independent runs with *K* from 1 to 8, each run with a burn-in period of 50,000 iterations and 300,000 Monte Carlo Markov Chain (MCMC) iterations, assuming an admixture model and correlated allele frequencies. The STRUCTURE data was subsequently analyzed by HARVESTER v.06.92 [39]. Mantel tests were made to determine if there are significant correlations between the dendrogram representations and the distance matrices, between the matrices of geographic and genetic distances, and between the distance matrices from the two DArT platforms used in the *P. fendleri*. The missing geographic coordinates of collection sites of ten accessions (PI 293027, PI 293028, PI 337050, PI 345712, PI 355037, PI 355041, PI 355042, PI 275771, PI 283700 and PI 299412) in the GRIN database were estimated using Google Earth v. 6.1.0.5001 based on available locality description and collectors’ notes and the information included in the analysis. Map projections of the analyzed *P. fendleri* accessions were made using ArcGIS Explorer v.2.0.0.1750 (ESRI, Redlands, CA) and non-parametric correlation tests done using JMP v.9 (SAS Institute, Cary, NC).

## Results

### Relationships among Accessions from Microarray DArT Analysis

**Figure 1:**
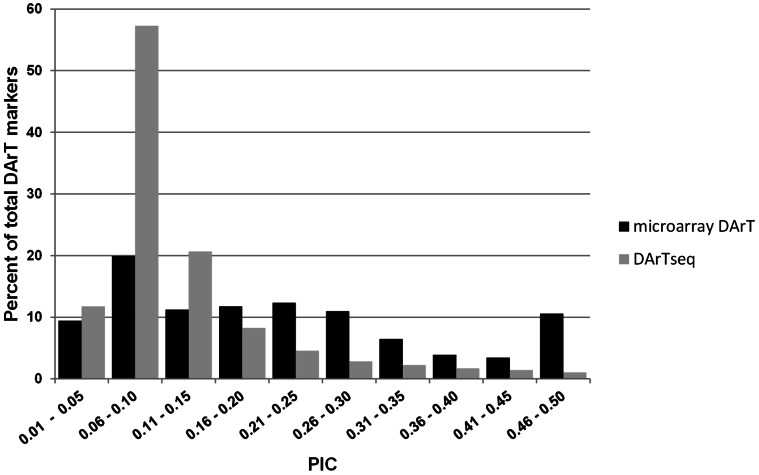
Distribution of marker PIC values by DArT platform.

A total of 2,833 polymorphic markers were found using microarray DArT, with an average genotype call rate of 98.4% and a scoring reproducibility of 99.7%. The average PIC value was 0.21 and the median 0.19. About 20% of the markers have values in the range of 0.06 to 0.10, and almost an equal proportion of 12% of markers on the following PIC classes - 0.11 to 0.15, 0.16 to 0.20, and 0.21 to 0.25 (Figure 1). Overall, the distribution of PIC values was asymmetrical and skewed towards the lower values.

**Figure 2:**
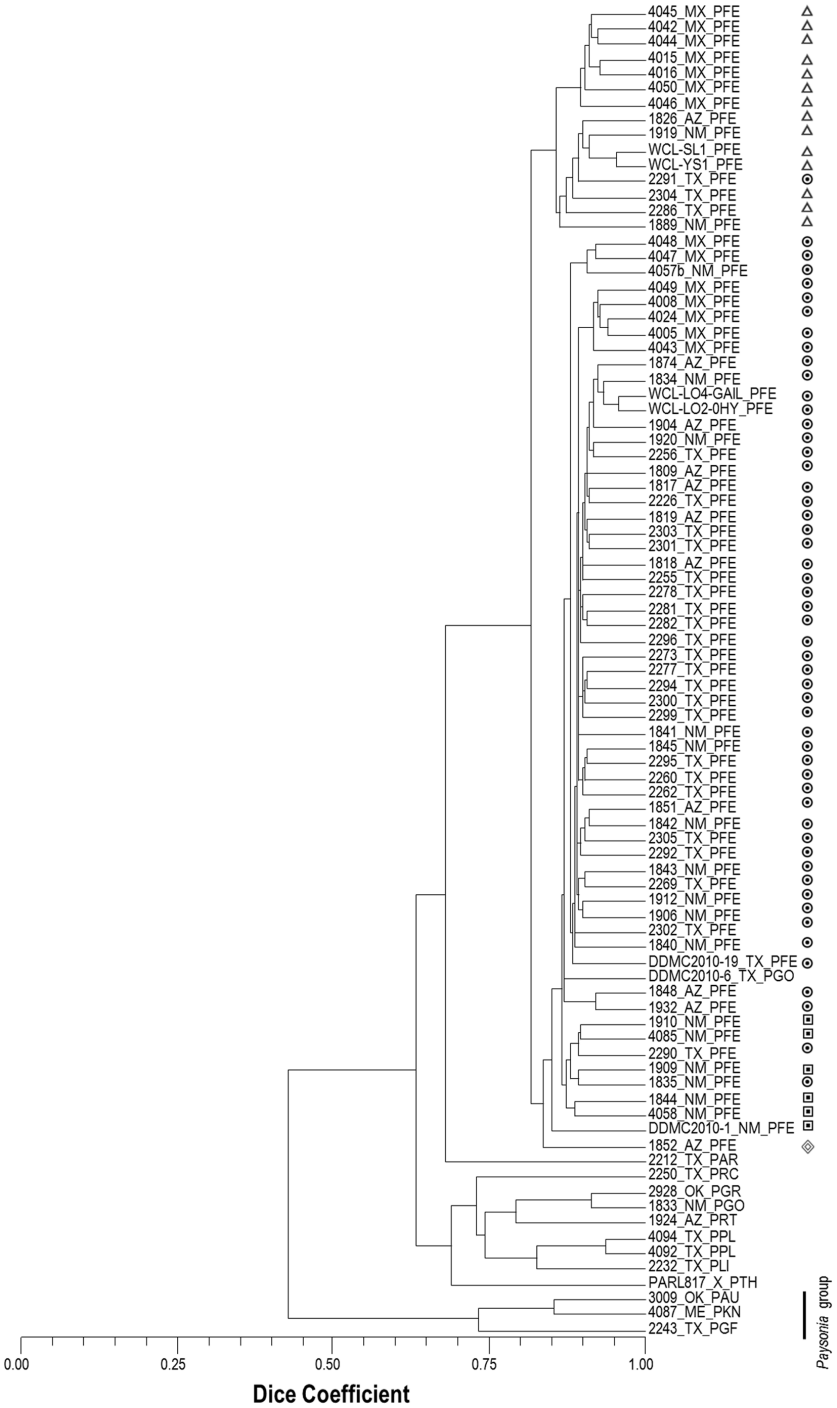
Cluster analysis of *Physaria* and *Paysonia* accessions based on 2,833 DArT markers. The labels denote the germplasm collection numbers and origin. Suffixes indicate respective species (PAR: *P. argyraea*, PAU: *P. auriculata*, PFE: *P. fendleri*, PGO: *P. gordonii*, PGF: *P. grandiflora*, PGR: *P. gracilis*, PKN: *P.* ‘kathryn’, PLI: *P. lindheimeri*, PPL: *P. pallida*, PRC: *P. recurvata*, PRT: *P. rectipes*, and PTH: *P. thamnophila*).

Cluster analysis and principal coordinate analysis (PC plot not shown) indicated that the different accessions were successfully classified by the marker system based on species, by geographical source, and breeding status (Figure 2), except for one new collection of *P. gordonii*, DDMC2010-6, which clustered with the *P. fendleri* accessions. The cophenetic correlation coefficient between the dendrogram and the distance matrix was highly significant (r = 0.98, t = 10.30, prob random Z<obs. Z = 1.00, 3000 permutations) indicating that the tree is a very good representation of the distance matrix. All *P. fendleri* accessions grouped in a separate cluster from the other species. The main cluster has two subgroups from Mexico, and a subgroup with a majority of accessions from Texas. There was no single group of *P. fendleri* accessions from Arizona and New Mexico. Most accessions from these States were associated with other accessions from Texas.

The four advanced *P. fendleri* breeding lines were partitioned into two clusters. The breeding lines WCL-SL1 and WCL-YS1 were found to be more genetically distant to WCL-LO2 and WCL-LO4. The last two lines were determined to be more similar to the rest of the *P. fendleri* from North America than WCL-SL1 and WCL-YS1.

Among the different species, the most similar to *P. fendleri* was determined to be *P. argyraea* while the least similar was *P. thamnophila*. The *P. pallida* accessions grouped in one cluster along with *P. lindheimeri* and other accessions representing the species, *P. gracilis*, *P. gordonii*, *P. recurvata*, and *P. rectipes*. The two accessions of *Paysonia auriculata* (3009) and *Paysonia grandiflora* (2243) grouped together in a separate cluster along with the interspecific hybrid swarm ‘Kathryn’ (4087) from five *Paysonia* species.

**Figure 3:**
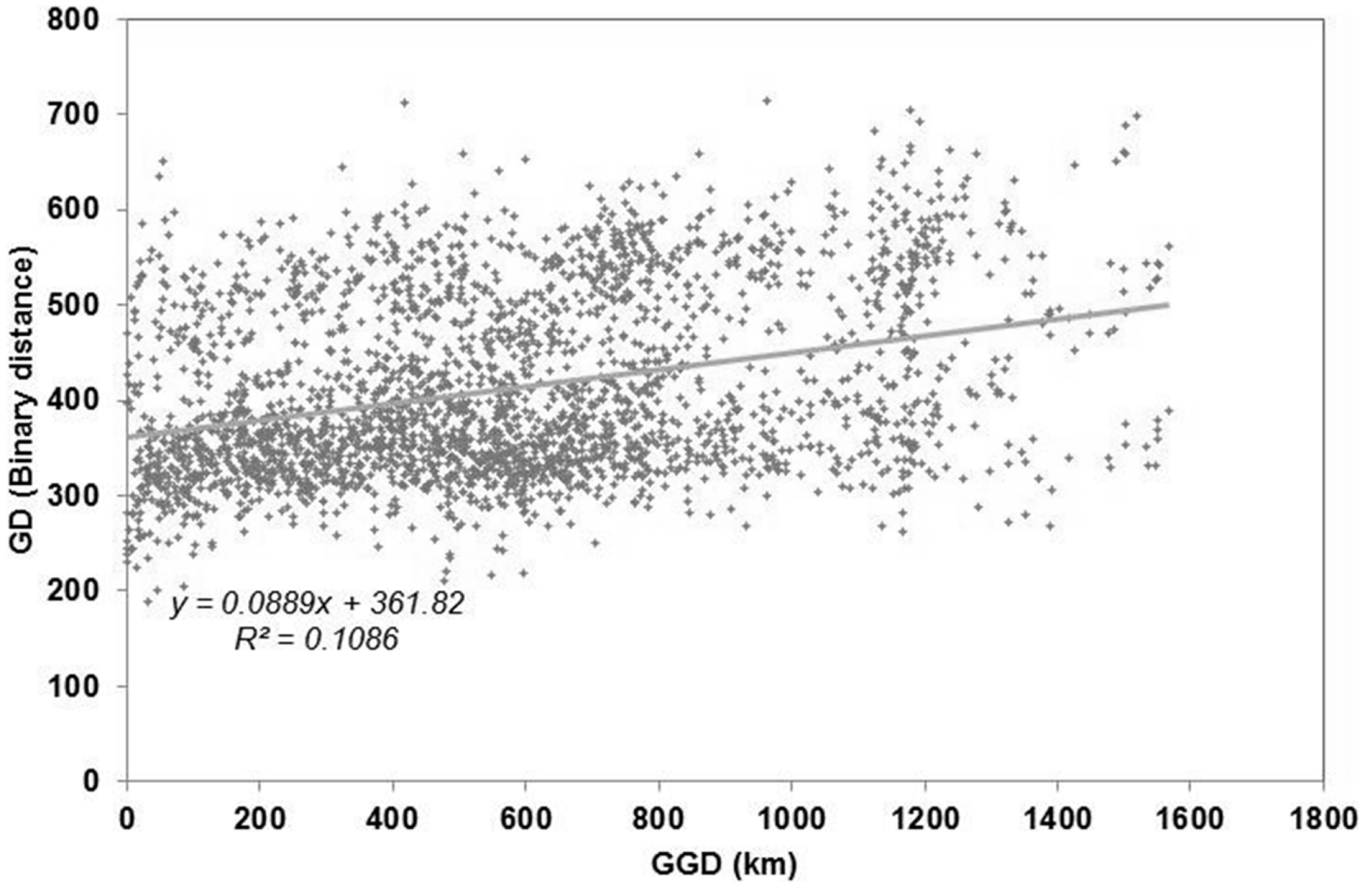
GenAlEx plot of geographic distance (GGD) and genetic distance (GD) from microarray DArT markers.

The analysis of molecular variance considering *P. fendleri* accessions only, showed that there was a much greater proportion of variation within groups (90%) than among groups (10%) in the species (Table 3). Among the unimproved germplasm set, the pairwise Fst values that showed the greatest differentiation was between Arizona accessions and those from New Mexico and the least amount of differentiation between Mexico and Arizona (Table 4). There was a significant correlation found between the computed genetic distance and geographic distance matrices from Mantel test (r = 0.33, t = 5.94, prob random Z<obs z = 1.00, 3000 permutations), indicating that distant accession pairs are more different genetically, supporting the previously mentioned result (Figure 3).

**Table 3:** Analysis of molecular variance in *P. fendleri* accessions.

Source	df	SS	Variance components	%
**A. Microarray DArT**				
Among Groups	4	1597.506	17.19956	9.66%
Within Groups	69	11094.561	160.79074	90.34%
Total	73	12692.068	177.99031	100%
**B. DArTseq**				
Among Groups	4	14299.235	98.59559	7.22%
Within Groups	123	155895.234	1267.44093	92.78%
Total	127	170194.469	1366.03651	100%

**Table 4:** Comparison of population pairwise Fst values using microarray DArT and DArTseq.

	Arizona	Advancedlines	Mexico	New Mexico	Texas
Arizona		**0.10717**	**0.13076**	**0.03055**	**0.04049**
Advanced lines	**0.13854**		**0.22289**	**0.10951**	**0.08193**
Mexico	0.00448	**0.13043**		**0.12926**	**0.10552**
New Mexico	**0.16823**	**0.17758**	**0.16280**		**0.03056**
Texas	0.01547	**0.12599**	**0.02649**	**0.16289**	

* Values in bold are significant at α = 0.05. Values below diagonal are from microarray DArT and those above diagonal are from DArTseq.

### Relationships among Accessions from DArTseq Analysis

There was a total of 27,748 markers obtained using the *Physaria* DArTseq platform. The average genotype call rate was 98.8% and a scoring reproducibility of 99.7%. The average PIC value was 0.12 and the median was 0.09. About 57% of markers have PIC values in the range of 0.06 to 0.10, while 20% and 11% on PIC classes 0.11–0.15 and 0.01–0.05, respectively (Figure 1). The `distribution of the PIC values of DArTseq markers follows the same skewed pattern as the DArT microarray markers presented earlier.

Cluster analysis using the markers from this platform resulted in a relationship that follows that of the taxonomic groupings based on general morphological affinities presented by Rollins and Shaw [1]. The results did not deviate from those when the first platform with fewer markers used. Four accessions of *P. fendleri* (2258, 2274, 3083, and DDMC2010-8) clustered with the other *Physaria* species – *P. gordonii* and *P. gracilis*, indicating higher genetic similarity to representative accessions of these species than the rest of *P. fendleri*. These four *P. fendleri* accessions will be further examined for misidentification and for oil and morphological trait variation when verified.

**Figure 4:**
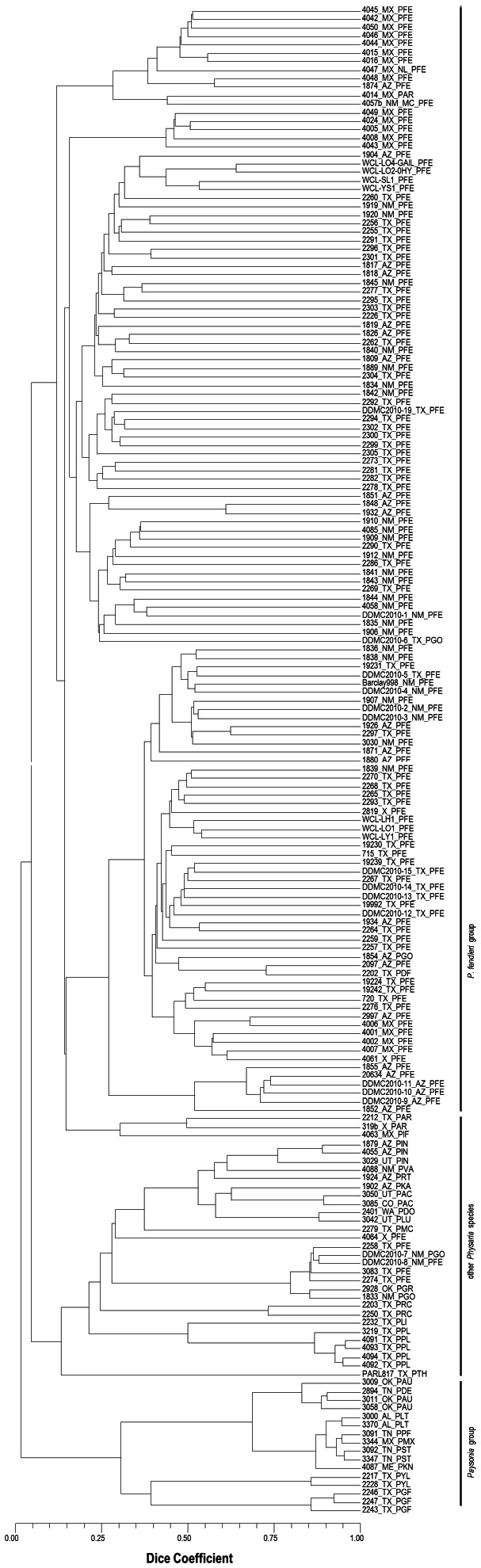
Cluster analysis of *Physaria* and *Paysonia* accessions based on 27,748 DArTseq markers. The labels denote the germplasm collection numbers and origin. Suffixes indicate respective species (PAC: *P. acutifolia*, PAR: *P. argyraea*, PAU: *P. auriculata*, PDE: *P. densipila*, PDF: *P. densiflora*, PDO: *P. douglasii*, PFE: *P. fendleri*, PGO: *P. gordonii*, PGF: *P. grandiflora*, PGR: *P. gracilis*, PIF: *P. inflata*, PIN: *P. intermedia*, PKA: *P. kaibabensis*, PKN: *P.* ‘kathryn’, PYL: *P. lasiocarpa*, PLI: *P. lindheimeri*, PLT: *P. lyrata*, PLU: *P. ludoviciana*, PMC: *P. mcvaughiana*, PMX: *P. mexicana*, PPF: *P. perforata*, PPL: *P. pallida*, PRC: *P. recurvata*, PRT: *P. rectipes*, PST: *P. stonensis*, PTH: *P. thamnophila* and PVA: *P. valida*).

The cophenetic correlation coefficient between the dendrogram and the distance matrix was highly significant (r = 0.94, t = 49.20, prob random Z<obs. Z = 1.00, 3,000 permutations) indicating that the tree is a very good representation of the distance matrix. The cluster of *P. fendleri* showed a distinct group of accessions from Mexico and a cluster comprised of all other germplasm from North America (Figure 4). The DArTseq platform indicated two separate clusters for the advanced lines. The breeding lines WCL-LH1, WCL-LO1, and WCL-LY1 are all in a group with greater similarity to accessions from Texas. WCL-LO2, WCL-LO4, WCL-SL1, and WCL-YS1 grouped together in one cluster indicating greater genetic similarity to accessions from Arizona and Mexico. WCL-LO2 was derived from the WCL-LO1 and the remaining three from WCL-LO2. It appears from these data that perhaps more genotypes from the Arizona accession were integrated into WCL-LO2.

Similar to results from the microarray DArT, the accessions of the following species formed distinct groups consistent with the classification by Rollins and Shaw [1]: a) *P. intermedia*, *P. valida*, and *P. rectipes*, b) *P. gordonii*, *P. gracilis*, *P. rectipes*, and *P. lindheimeri*, and c) *P. auriculata*, *P. densipila*, *P. lyrata*, *P. stonensis*, *P. grandiflora*, and *P. perforata*. The two accessions of *P. lasiocarpa* (2217 and 2228) were most genetically similar to the accessions of *P. grandiflora.* This last group of species comprised of annual, auriculate-leaved types whose taxonomic nomenclature was segregated from *Physaria* and transferred to *Paysonia* based on leaf-trichome morphology, chromosome number, and molecular data from analyzing internal transcribed spacers of nuclear ribosomal DNA [40]. Apart from the accession of *Physaria mexicana* nested within the *Paysonia* cluster, the results of this genetic analysis using both microarray DArT and DArTseq platforms support the previous segregation of *Physaria* from *Paysonia* as proposed by O’Kane and Al-Shehbaz [40] indicating that the group of *Paysonia* species to be the least genetically similar to the *P. fendleri* and other *Physaria* accessions.

Results of the analysis of molecular variance when DArTseq markers were used correspond to that from microarray DArT. A greater proportion of variation within groups (93%) than among groups (7%) in the species was found (Table 3).

The average genetic similarity in the *P. fendleri* group when using microarray DArT was 0.86, while only 0.44 when DArTseq was used. This is in line with the assumption that more differences may be found when more markers are used because of the increased sensitivity and resolution to detect genetic distinctiveness [41]. The genetic similarity matrices of *P. fendleri* obtained using the two platform systems showed a good fit when compared by a Mantel test (r = 0.48, t = 11.28, prob random Z<obs z = 1.00, 3,000 permutations).

### Population Structure Analysis of *P. fendleri*

**Figure 5:**
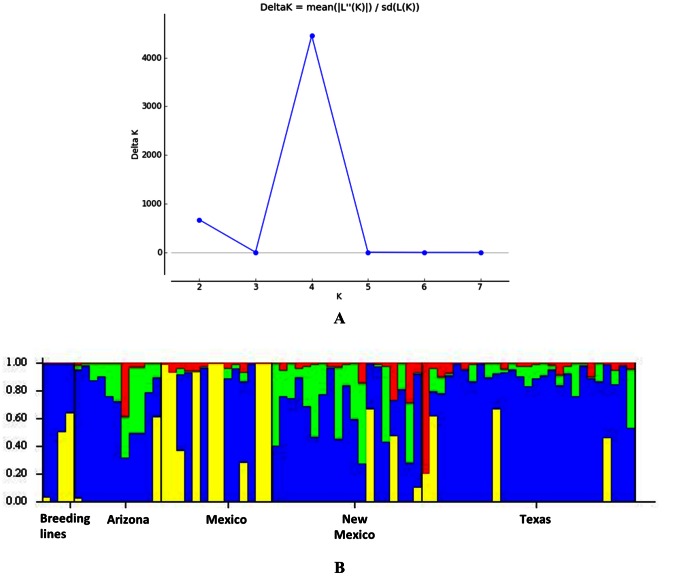
Plot of Δ*K* from K = 2 to 7 (a) and the population structure of 75 *P. fendleri* accessions at *K* = 4 (b).

**Figure 6:**
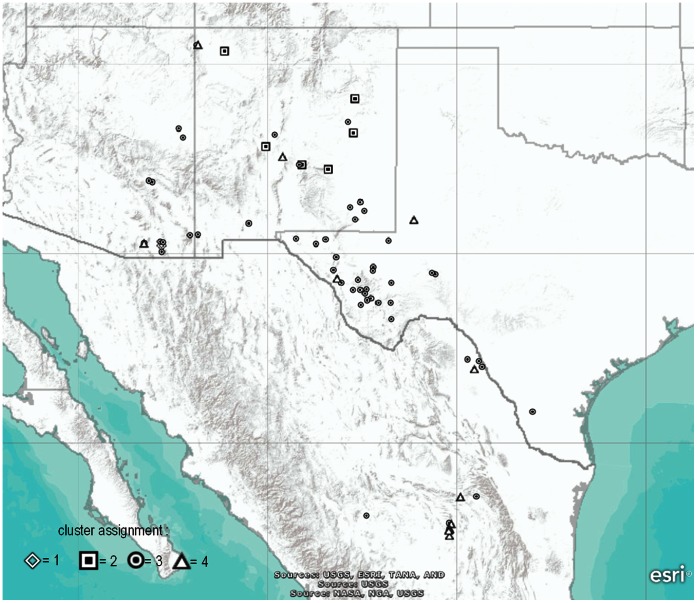
Geographic locations of the *P. fendleri* accessions and their respective cluster assignments (indicated by different icons) based on Bayesian model-based clustering methods.

Using results from microarray DArT, the population structure of the *P. fendleri* samples was determined. The plot of Δ*K* for each *K* value is shown in Figure 5a. It was estimated through the method of Evanno et. al. [42] that there are 4 groups contributing significant genetic information in the *P. fendleri* collection. The bar plot of the population assignment test when *K* = 4 is shown in Figure 5b. Three of the four accessions of *P. fendleri* breeding lines are shown to have mixed backgrounds. Of the other *P. fendleri* accessions, twelve (16%) have close to homogeneous genetic background (>98% probability) while 63 accessions (84%) are highly heterogeneous showing intermediate and/or highly mixed composition. A majority of the accessions from Texas are assigned to one cluster, while those from New Mexico have myriad cluster assignments suggesting the greater diversity of *P. fendleri* in this U.S. state. When the cluster assignments of the *P. fendleri* accessions were projected on a map, the segregation among clusters was evident in their geographic location (Figure 6). Further testing for association between the assigned clusters and available site elevation data by computing Spearman’s rank correlation coefficient showed a very weak positive correlation (Spearman ρ = 0.11, p = 0.33).

## Discussion

The importance of understanding genetic diversity in germplasm collections is critical for the effective management of accessions in genebanks. Molecular characterization supplements morphological evaluation of germplasm and allows measurements to help resolve numerous operational, logistical, and biological questions that face genebank managers and conservation biologists [43], [44]. The *Physaria* collection in the U.S. NPGS has not been well characterized before for genetic diversity, though there have been preliminary studies on subsets of accessions using a limited number of microsatellite markers [16], and an extensive evaluation for diversity in oil characteristics and other morphological characters [45]. In this study, the new DArT platforms for *Physaria* were found both acceptable and provided robust information about the genetic variability of the collection.

The development and utilization of DArT markers allowed us to determine the genetic diversity of the *Physaria* collection. The 2,833 microarray DArT markers were found to be useful in providing a picture of genetic diversity in the *Physaria* germplasm collection using a large set of accessions. Overall, the average PIC of the *Physaria* and *Paysonia* microarray DArT markers was found to be lower than that observed in other species where similar markers were developed, like wheat (0.44) [46], cassava (0.42) [47], and sorghum (0.41) [48], but comparable to that observed in sugar beet (0.28) [49] and *Asplenium* fern (0.21) [50]. The average PIC of the DArTseq markers is much less than that of the microarray DArT. However, the more numerous DArTseq markers may have the capability of providing a better picture of diversity by sampling more points in the genome. The distribution of these almost 30,000 new DArT markers in the *Physaria* and *Paysonia* genome remains to be determined. However, based on the information from a large number of organisms in which DArT system was applied more broadly including genetic mapping and/or sequence-based physical mapping, we can assume that DArT marker from both platforms will be distributed throughout the genome with marker density highly correlated to gene density [28], [51]. Compared to microsatellite markers, DArT markers are very suitable for high-throughput work and previously have been determined to have clear advantages in cost and time aspects of genotyping as demonstrated in other crops [52]. Both microarray DArT and DArTseq platforms have the same development costs. However, the higher number of markers obtained in DArTseq resulted to an overall lower cost per datapoint than microarray DArT. This higher cost effectiveness of DArTseq is in parallel to other sequenced-based genotyping strategies which can provide substantial cost savings compared to microarrays when conducting genetic diversity studies [53], [54]. Importantly, when comparing *effectiveness* of the two platforms one has to keep in mind that it may vary according to specific *application*: in genetic ID and product quality testing (i.e. seed purity) modest number of array-based DArT markers may perform as well as DArTseq platform and currently for a better price. A further cost reduction of sequencing may however push even this balance towards DArTseq platform in the future.

The relationships found among the accessions are in line with the previously proposed evolution within the genus. Taxonomists assert that *P. auriculata* is the most primitive species due to its very distinct evolutionarily primitive characters such as large siliques, large number of ovules around the replum, and predominance of simple trichomes [1]. *P. auriculata* has been proposed to be closely related to *P. grandiflora* and this relationship is supported by the results of the molecular marker analysis between the accessions representative of these species (2243 and 3009) showing high genetic similarities. Likewise, *P. gordonii* and *P. gracilis* grouped in the same cluster with *P. rectipes*, *P. recurvata*, and *P. thamnophila* which is in agreement with their previously known taxonomic groupings based on very general morphological similarities. The high genetic similarity between the *P. argyraea* accessions (2212 and 319 b) to the *P. fendleri* group supports phylogenetic subsectional grouping based on pod morphology.

Overall, there was higher genetic similarity found among accessions of the other *Physaria* and *Paysonia* species than among accessions of *P. fendleri*. In particular, species that are included in the federal or state threatened and endangered species list, like *P. pallida* and *P. stonensis* [55], have very low genetic diversity as indicated by results of DArTseq markers. In *P. pallida*, the representative accessions (4091 and 4093) were found to be highly similar. The limited geographic range and the proximity of the collection sites of these two samples suggest that they might have been from just one population. Likewise for *P. stonensis*, there was a very high genetic similarity found on both of the representative accessions (3092 and 3347). Because only a limited number of accessions were included in these other species, a follow up study that includes additional accessions is recommended to validate if this is a general trend.

*Physaria ‘*Kathryn’ (4087) is a cultivar from interspecific hybridization developed by allowing five species (*P. densipila, P. lescurii, P. lyrata, P. perforata,* and *P. stonensis*) to intermate for twenty two generations [56]. *P. ‘*Kathryn’ was determined to be most closely related to *P. auriculata* in our analysis using microarray DArT. However, with the expanded set of accessions included in the DArTseq analysis, it grouped with the representative samples of its parent species – *P. lyrata* (3000 and 3370), *P. perforata* (3091), and *P. stonensis* (3092, 3347). These species were also found most genetically similar to *P. mexicana* (3344) which is the only perennial type in the species cluster. *P. mexicana* is a previously undescribed species in Mexico and is among the more recent species reported by Rollins [57].

DDMC2010-6 was from a more recent germplasm collecting trip and it was entered in the database as *P. gordonii*. However, based on results of DArT markers, it clustered with the *P. fendleri* accessions after using both DArT platforms, indicating the need to review its species assignment. The species identity of this particular accession will again be verified using its plant voucher specimen as well as in the NPGS site handling the germplasm when it is regenerated in the future.

The two clusters of *P. fendleri* breeding lines - WCL-LO2, WCL-LO4, WCL-SL1, and WCL-YS1 in one cluster, while the other set of breeding lines WCL-LO1, WCL-LH1, and WCL-LY1 in another, indicates the possibility of developing genetically differentiated lines for crop improvement and may have applications in future hybrid development work. The lines WCL-LH1, WCL-LO1 and WCL-LY1 are the first three germplasm lines that were publicly released in 1996. These were developed using recurrent selection on a population made by bulking seeds of one accession that came from Arizona and nine from Texas in 1986 [58]. The bulked seeds were also the starting material for other breeding lines. WCL-LO4 was derived from mass selection from WCL-LO3 and WCL-LY2 (both not included in this study) which has WCL-LY1 as the source population [59], [60]. The other breeding lines were developed through phenotypic selection: WCL-YS1 was a selection from PI 311165, one of the initial accession from Arizona that comprise the original bulk in 1986 [61], while WCL-SL1 came from plants that survived at the highest salinity levels during a salt tolerance screening study when seeds from the original bulked seeds were planted [62].

The accessions of *P. fendleri* from Mexico are genetically similar as indicated by the cluster analyses. The range of *Physaria* species has been reported as limited to the northeastern part of country and concentrated on mountain and high plains of Coahuila, Nuevo León, and Zacatecas [57]. This limited geographic distribution likely prevented their further genetic differentiation. A similar investigation focusing on the other *Physaria* species in this region could confirm this trend.

The array of *P. fendleri* germplasm consisted of 75 accessions analyzed using microarray DArT and 128 accessions using DArTseq. There is ample genetic variability in the *P. fendleri* collection found, as indicated by the cluster analysis as well as analysis of population structure. This is expected for a cross pollinating species that has not been fully domesticated [63]. However, the higher within group variation detected by AMOVA using data from both DArT platforms suggests that there is only a small amount of genetic differentiation among groups in the sample as a whole. Results from the Bayesian clustering approach when comparing the geographical sources of the accessions suggested that there is more variability in New Mexico than the other *P. fendleri* locations and that there is a spatial pattern evident in the microarray DArT results. This pattern of genetic differentiation occurs outward from central Texas, the region identified as the putative origin of *Physaria* as proposed earlier by Payson [64].

An increased population differentiation has been reported in many plant species between source populations and new ones when plants colonize new habitats [65]. The more dynamic nature of *Physaria* populations in distant locations from Texas has been reported by Payson [64] and he attributed part of the process as caused by barriers that separate populations, such as soil properties and moisture availability which are very important to survival of ephemeral populations of the taxon. In *P. fendleri*, Dierig et al. [66] stated that temperature and elevation effects can also account for significant differences in reproductive capacity. Selection patterns have also been investigated by past studies reporting that non-random mating, sexual selection and dormancy characteristics play a significant role in how traits evolved in the species [67], [68]. Seed dormancy characteristics in particular cause a persistent soil seed bank which may prevent genetic differentiation in the species because certain genotypes are reintroduced back during subsequent seasons [69], [70]. In terms of genetic resources conservation of *P. fendleri* germplasm, the DArT results suggest the existence of more variable genotypes in New Mexico and it is recommended that future collecting missions select this geographic area to possibly expand the genetic diversity in the species collection.

### Conclusion

The availability of genetic diversity information of the *P. fendleri* collection will enable better germplasm management and conservation of the species. In this study we report the successful development of two DArT marker platforms that were utilized for genotyping *Physaria* and *Paysonia* accessions. This marker system complements the microsatellite markers developed previously for *Physaria*. The high number of DArT markers allows a greater resolution of genetic differences among accessions and enabled us to examine the extent of variation in the *P. fendleri* collection, as well as provide support to known taxonomic classification and recent nomenclatural changes of certain *Physaria* species to *Paysonia.* We intend to further utilize the DArT markers in developing a linkage map in *Physaria* to assist breeding efforts and for future genetic mapping studies.
